# Bovine pericardium based non-cross linked collagen matrix for successful root coverage, a clinical study in human

**DOI:** 10.1186/1746-160X-8-6

**Published:** 2012-03-05

**Authors:** Markus Schlee, Shahram Ghanaati, Ines Willershausen, Michael Stimmlmayr, Anton Sculean, Robert A Sader

**Affiliations:** 1 Private Practice. Bayreuther Strasse 39, 91301 Forchheim, Germany; 2 Department of Oral, Cranio-Maxillofacial and Facial Plastic Surgery, Medical Center of the Goethe University Frankfurt, Germany; 3 Institute of Pathology, Johannes Gutenberg Universität Mainz, Germay; 4 Department of Operative Dentistry, University Medical Centre of the Johannes Gutenberg University Mainz, Germany; 5 Private Practice, Josef-Heiligenbrunner-Str.2, 93413 Cham; 6 Department of Periodontology, University of Bern, Bern, Switzerland

**Keywords:** Gingival recession, root coverage, collagen matrix, guided tissue regeneration, bovine pericardium, connective tissue

## Abstract

**Introduction:**

The aim of this study was to clinically assess the capacity of a novel bovine pericardium based, non-cross linked collagen matrix in root coverage.

**Methods:**

62 gingival recessions of Miller class I or II were treated. The matrix was adapted underneath a coronal repositioned split thickness flap. Clinical values were assessed at baseline and after six months.

**Results:**

The mean recession in each patient was 2.2 mm at baseline. 6 Months after surgery 86.7% of the exposed root surfaces were covered. On average 0,3 mm of recession remained. The clinical attachment level changed from 3.5 ± 1.3 mm to 1,8 ( ± 0,7) mm during the observational time period. No statistically significant difference was found in the difference of probing depth. An increase in the width of gingiva was significant. With a baseline value of 1.5 ± 0.9 mm an improvement of 2.4 ± 0.8 mm after six month could be observed. 40 out of 62 recessions were considered a thin biotype at baseline. After 6 months all 62 sites were assessed thick.

**Conclusions:**

The results demonstrate the capacity of the bovine pericardium based non-cross linked collagen matrix for successful root coverage. This material was able to enhance gingival thickness and the width of keratinized gingiva. The percentage of root coverage achieved thereby is comparable to existing techniques. This method might contribute to an increase of patient's comfort and an enhanced aesthetical outcome.

## Introduction

According to the Glossary of Periodontal Terms (2001), gingival recessions are defined as "location of marginal periodontal tissues apical to the cemento-enamel junction " [1]. It is well known that the major causative factors in the development of marginal tissue recessions are a bucal/lingual malposition of the tooth, a thin gingival biotype, dehiscence and fenestration of the bucal alveolar bone and iatrogenic factors such as orthodontic treatment. A possible influence of occlusal trauma is still discussed. Localized gingival recessions and root exposure may cause an aesthetic problem for the patient and are often associated with dentine hypersensitivity [2]. Further, root caries and persisting gingival inflammation are frequently observed. All of these situations represent an indication for regenerative periodontal therapy. In these cases the coverage of the recession is highly beneficial as it will facilitate plaque control, improve aesthetics and reduce root sensitivity. While complete root coverage can be achieved in class I and II defects, only partial coverage may be expected in class III. Class IV defects are not amenable for coverage. Consequently, in recessions that are suitable for coverage, the critical decisive clinical variable to estimate the possible outcome of root coverage procedures is the level of periodontal tissue support at the proximal surfaces of the tooth.

Various root coverage procedures have been used to cover localized gingival recessions, such as laterally repositioned flaps [3-5] coronally advanced flaps [6] free gingival grafts [7,8] connective tissue pedicle flaps with a free gingival graft subepithelial connective tissue grafts [9-14] acellular human dermal matrix allografts [15] and guided tissue regeneration [16].

Collagen membranes, mostly bovine and porcine derived collagen types 1 and 3 [17] are the membranes most frequently used in clinical routine. The ability of collagen to promote progenitor cell adhesion, chemotaxis, homeostasis and physiological degradation, together with easy manipulation and low immunogenicity makes it an ideal material for barrier preparation. Collagen is degraded by matrix metalloproteases (MMPs) [18]. During wound healing, neutrophils, monocytes, and fibroblasts release MMPs to the wounded area, thus contributing to collagen membrane degradation. Some advantageous properties of collagen over other materials include hemostatic function, allowing early wound stabilization. Its chemotactic properties attract fibroblasts and their semi permeability allows nutritient transfer [19]. Native collagen, however, has been reported to undergo a fast biodegradation. Common approach to slow down degradation is by increasing the amount of collagen cross liking by means of ultraviolet and γ radiation, hexamethylenediisocyanate, glutaraldehyde, diphenylphosphorilazide, and ribose have been successful in achieving collagen cross-linking [20,21]. Chemical cross-linking, however, has been reported to inhibit the attachment and proliferation of osteoblasts and fibroblasts [22].

In an in vivo study *Ghanaati et al*. proved that a non-cross linked porcine derived collagen types 1 and 3 matrix i.e. Mucograft^® ^remains stable as matrix after its well integration within the subcutaneous tissue of mice and human [23]. However, only a mild vascularization of both, the matrix and its implantation bed was observed in both species.

It seems that non-cross linked porcine derived collagen types 1 and 3 are well integrated in the implantation bed and are able to contribute to soft tissue augmentation by undergoing integration and only a slow physiological biodegradation without the induction of a foreign body response [23]. Accordingly, all non-cross linked collagen type I and III-based matrices, independent of the origin of the species, i.e. bovine or porcine with compositions similar to Mucograft^® ^might bear the above-mentioned capacity to augment and enhance the soft tissue. To assess that, a new non-cross linked bovine pericardium derived collagen type I was clinically used, in order to evaluate its contribution to the periodontal plastic surgery. In this study a new method of increasing gingival thickness by implantation of acellular deproteinised soluble degraded bovine pericardium (Copios^®^) was used. Coverage, thickness, clinical attachment level and width of keratinized gingiva were examined.

## Materials and methods

### Copios^®^

**Figure 1:**
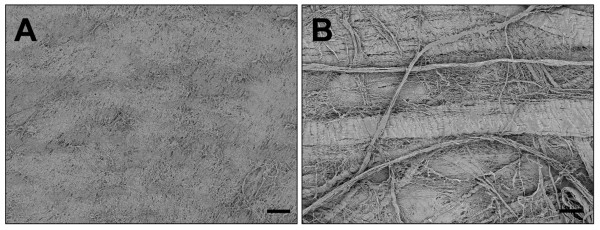
**Shows both sites of the acellular deproteinised bovine pericardium (Copios^®^); 100 × magnification, scale bar = 100 μm**. A) shows the plain site. B) shows the rough site.

In the present study a matrix derived from bovine pericardium, Copios^® ^(Zimmer, Freiburg, Germany) was employed. This matrix was processed according to the Tutoplast manufacturing methodologies. The Tutoplast^® ^process is a chemical method, originally developed more than 30 years ago for sterilization and preservation of tissue intended for implantation. The Tutoplast^® ^process combines osmotic, oxidative and alkaline treatment of the tissues in order to break down cell walls, inactivate pathogens, and remove bacteria. The pericardium is delipidizated in an ultrasonic bath with acetone. Alterning washing in NaCl, H_2_O_2 _and acetone breaks down the cellular walls, removes fat and proteins, dehydrates, inactivates and eliminates viruses and prions. The product is sterilized with a low-dose gamma irradiation (17.8 kGy) according SAL 10-6, AAMI Standard, EN 552 and CE approved. Prior to usage, the material needs to be rehydrated with sterile saline solution. By means of this procedure, the original cross linking and the 3-D structure of this type I collagen stays unchanged. Scanning microscopy images of the smooth and the rough sites of the membrane are displayed in Figure 1.

### Inclusion criteria and subject and site selection

In the present case report, which was approved the ethical commission( Freiburger Ethikkommission International, FEKI (study code ZD-MS-2011-01)) a collective of fourteen systematically healthy patients (n = 10 female, n = 4 male), non-smokers for at least 6 months, with a total of 62 Miller Class I or II recessions were selected from the patient pool of a private practice. The age of the patients ranged between 34 and 59 years (mean age 45.3 years). In order to be included in this study, every patient had to display at least one buccal recession classified as Miller Class I or II, with no visible loss of interdental soft and hard tissue. Criteria for inclusion in this study were systemic health, no permanent medication within the last six months, no mental illness, physical disabilities, neuropathy or severe CVD. Further oversensitivity to bovine products and smoking served as exclusion criteria. All subjects who were included in this examination gave informed consent to participate. Following an initial examination the patients were instructed in oral hygiene, and professional tooth cleaning was performed. Plaque and bleeding scores had to be ≤ 15% prior to surgery. Plaque and bleeding scores were documented as the percentage of total surfaces (6 aspects per tooth). Positive and/or negative plaque and bleeding scores were recorded. The bleeding scores were used to evaluate the degree of inflammation. All measurements were carried out with a pressure sensitive periodontal probe (PCP-UNC 15 probe, Hu-Friedy) with a calibrated force of 0,3 N to the nearest 0,5 mm. To avoid intra-examiner errors, all soundings were performed by one calibrated investigator only.

### Surgical procedures

**Figure 2:**
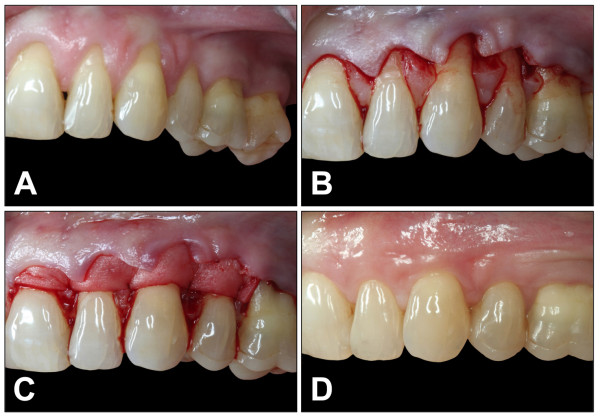
**Illustrates the surgical procedures of a coronal advanced flap and the implantation of the acellular deproteinised bovine pericardium (Copios^®^) for soft tissue regeneration in human**. A) Multirecession within the upper jaw with thin biotype and inserting frenula. B) Incision outline as described by Zucchelli. C) Bovine pericardium is fixed with sling sutures arround the teeth. D) Six months after implantation: Biotype and width of keratinized gingiva is visibly enhanced. The frenula insert deeper.

All surgeries were performed by the author (MS). After local anesthesia the exposed root was scaled and planed to the bottom of the pocket with rotating burs, ultrasonic instruments and curettes. Deeper instrumentation was avoided to prevent fibrous attachment. The root was flattened, smoothened and decontaminated. No chemical root conditioning was performed. After sulcular incision a coronal displaced split thickness flap without releasing incision, according to the incision outline described by Zucchelli, was performed. The flap preparation in difference to Zucchelli was a complete split thickness flap. The flap was considered mobile enough when it stayed passively at a level coronal to the cemento-enamele junction (CEJ). The above mentioned bovine pericardium derived membrane was used to enhance the thickness and stability of gingival tissue. Prior to implantation the material was rehydrated as on the dry matrix surface blood would immediately coagulate and consequently impair its integration within the tissue. The matix was adapted in size and fixed by sling sutures (Prolene, 6-0, Braun, Melsungen, Germany) around the recipient teeth. The matrix was trimmed to reach at least a level 2 mm apical the bony margin. The coronal position was 1 mm below the CEJ. The knots were placed on the lingual aspect. Painless and atraumatic removal is possible then. The coronal advanced flap completely covered the membrane and was also fixed by sling sutures to accomplish a precise adaptation around teeth. The steps of the surgical procedure are displayed in Figure 2.

### Postsurgical protocol

Patients were instructed to avoid any mechanical traumata of the wound. For 4 weeks tooth brushing in the respective area was not allowed. Plaque prevention was achieved by mouthrinsing with chlorhexidine solution (0,12%) twice a day for one minute. A nonsteroidal and anti-inflammatory analgentic, Ibuprofene, was prescribed. No antibiotics were recommended. Sutures were removed two weeks after surgery. Patients were enrolled in a recall at 4, 8 and 24 weeks after surgery, which included professional plaque control remotivation.

### Clinical measurements

At baseline, i.e. prior to the intervention and six months after successful surgery the following parameters were recorded and described as below: Gingival recessions (Rec) were measured from the cement-enamel-junction (CEJ) to the most apical position of the gingiva on the buccal aspect of the root. Probing depth (PPD) was measured from the most apical position of the gingiva on the buccal aspect of the root to the bottom of the gingival sulcus. The clinical attachment level CAL was calculated on the basis of the measured clinical data by means of the following formula: CAL = Rec + PPD. The width of keratinized gingiva (KG) was measured from the gingival margin to mucogingival junction (MGJ). The biotype (BT) of gingiva was assessed visually and either considered to be thick when the probe was not visible through the tissue and thin at sites where the probe was visible through the tissue. Percentage of root coverage (RC) was measured and calculated 6 months after surgery according following formula: RC = 100 × (Rec baseline - Rec 6 months): Rec baseline.

### Statistical analysis

Quantitative data are presented as mean ± standard deviation and statistical analysis was performed using SPSS 15.0 (SPSS Inc., Chicago, IL). Differences were considered significant if P-values were less than 0.05 (*P < 0.05), and highly significant if P-values less than 0.01 (**P < 0.01). The nature of this study is exploratory, so all p-values are descriptive.

## Results

**Figure 3:**
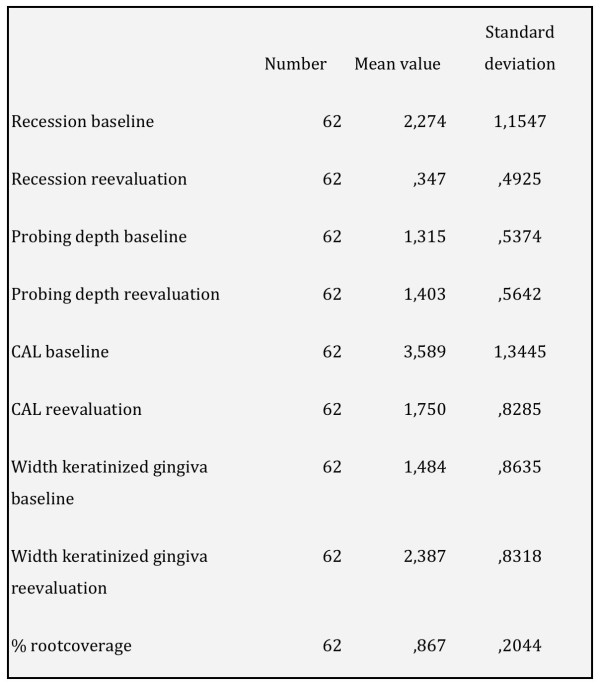
**Illustrates the clinical parameters at baseline and six months after implantation**.

**Figure 4:**
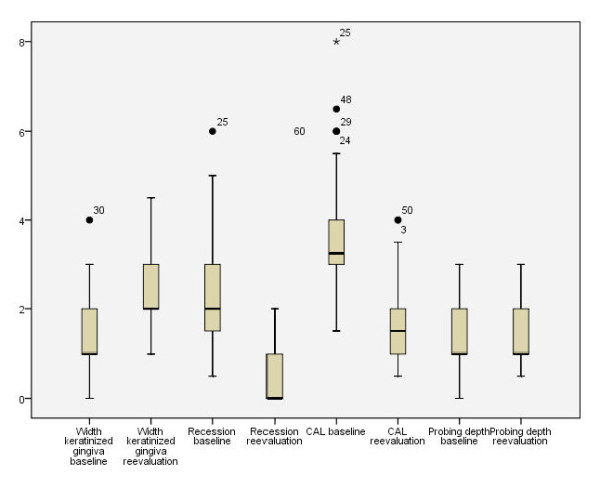
**Shows the box-blot-analysis of the investigated clinical parameters**.

All surgeries were performed without any complication. All patients tolerated the surgical approach and the used bio-membrane was well integrated without any visible local or systemic signs and symptoms of rejection. Within the observational period the clinical parameters were measured twice, at baseline and after six months, the results of which are described below. All patients fulfilled the protocol and attended all the follow-up visits. The mean number of treated recessions was 4.4 in every patient, this number varied from 1 to 11 recessions treated in a single patient. The mean percentage of root coverage measured six month after surgery was 86.7%. In 36 out of the 62 recessions treated, complete root coverage was obtained. The use of the novel bovine pericardium matrix led a to a highly significant reduction of the mean recession depth from 2.2 ± 1.1 mm to 0.3 ± 0.5 mm (p < 0.001). The total loss of clinical attachment (CAL) was initially measured with 3.5 ± 1.3 mm. This parameter could equally be significantly reduced, leading to satisfying values of 1.8 mm ± 0.7 mm after six months (p < 0.001). Also, the insertion of the Copios^® ^matrix increased the width of keratinized Gingiva from 1.5 ± 0.9 mm at baseline to 2.4 ± 0.8 mm. The change in biotype was one of the most remarkable aspects observed in this study. Six month after treatment all 62 recessions could be assessed to have a thick biotype, while only 22 recessions were considered to be thick at baseline. However, no change was observed when looking at the probing depth. After 6 month the mean pocket depth ranged at 1.4 ± 0.5 mm, while the initial pocket depth at baseline was measured at 1.3 ± 0.5 mm (p < 0. 213). The investigated clinical parameters and their changes over the observational time period are highlighted in Figure 3 and 4.

## Discussion

With the recent advances in modern dentistry, localized gingival recessions and root exposure represent an aesthetic problem to the discerning patient. Further, root caries, dentine hypersensitivity and persisting gingival inflammation are frequently observed. All of these situations represent an indication for periodontal plastic surgery. With this intervention being a highly elective one, treatment modalities have to focus on complete coverage of the exposed root, leaving no millimetre uncovered. Furthermore, a perfect match of colour and texture of tissues should be achieved. Coronally advanced flaps, laterally repositioned flaps, free gingival grafts, and subepithelial connective tissue grafts appeared as novel approaches to achieve improvements in recession depth, clinical attachment level and width of keratinized tissue [6,24,25]. Techniques such as free gingival grafts are reported to fail frequently [26,27]. Connective tissue grafts, however, are described to cause excessive tissue thickness as a post surgical complication [9-12]. The major drawbacks of this technique are bleeding of the donor site, sensations of pain and hyposensitivity of the palate [9-12]. In multi-recession cases connective tissue grafts can be applied only with retention as the amount of donor tissue limits the treatable area. Multiple surgeries have to be performed in those cases, imposing physiological and psychological stress on the patient. On the basis of these complications, a large number of biological biomatrices have been introduced within the last decades, all of which have been reported to contribute to clinically relevant root coverage [23,28,29]. These matrices were developed with the intention to reduce the major disadvantages of connective tissue grafts. When looking at the success rate, long term stability and aesthetical outcome every matrix has to face the comparison with the connective tissue graft. Due to its perfect colour match, the bilaminar blood supply and the high grade of clinical success this technique is still regarded as the gold standard. In the present study Copios^® ^(Zimmer, Germany), a matrix derived from bovine pericardium was used in order cover teeth with recession of Miller class I and II. The matrix was well tolerated by all of patients with no allergic reaction or other complications observed within the study period. The data of this study were able to prove that obviously, the application of the Copios^® ^matrix leads to a marked increase of the clinical attachment level after the observation time point of 6 month. These results are in accordance with previously published data employing a similar human dermal derived matrix [29]. In this study no change was observed in the probing depth within the six months. The width of gingiva increased in average 0,9 mm, which is slightly less compared to the values reported for connective tissue grafts. However, when comparing those values to AlloDerm products they are slightly higher [28]. This observation is also true for the width of keratinized gingiva and the gingival biotype.

The harmlessness of the use of Tutoplast, which is the basis of Copios^® ^has been previously described for application in dental surgery [29] as well as for closing ventricular septal defects in cardiovascular surgery [30] and dura replacement in neurosurgery [31]. Furthermore, animal studies in rats proved a complete integration of the membrane by recipients' connective tissue. Approximately 60% of the original membrane thickness remained [32]. The results underline that obviously, non cross linked collagen based matrices such the above mentioned material, show similar integrative capacities for soft tissue augmentation as observed for the CTG and other porcine derived matrices. The used matrix was able to be integrated within host connective and thereby augmenting the biotype by enhancing gingival thickness. However, further investigations with this material and with patients own connective tissue is necessary to critically assess the potential of this new membrane for a long-term clinical use.

## Summary

The results demonstrate the efficacy of the presented technique. Bovine pericardium acts as a scaffold and is integrated by recipient's connective tissue. This enhances gingival thickness. This method might contribute to an increase of patient's comfort and an enhanced aesthetical outcome. Even multi recession cases can be done in one stage.

## Competing interests

The authors declare that they have no competing interests.

## Authors' contributions

MS carried out the study and treated all patients. SG, IW and MS drafted and designed the manuscript. AS and RS were involved in designing and revising the manuscript. All authors read and approved the final manuscript.
